# A Worksite Nutrition Intervention is Effective at Improving Employee Well-Being: A Pilot Study

**DOI:** 10.1155/2018/8187203

**Published:** 2018-05-02

**Authors:** Jay T. Sutliffe, Mary Jo Carnot, Joel H. Fuhrman, Chloe A. Sutliffe, Julia C. Scheid

**Affiliations:** ^1^Nutrition and Foods, Northern Arizona University, Flagstaff, AZ, USA; ^2^Psychological Sciences, Chadron State College, Chadron, NE, USA; ^3^Nutritional Research Foundation, Flemington, NJ, USA; ^4^Northern Arizona University, Flagstaff, AZ, USA

## Abstract

**Background:**

Worksite dietary interventions show substantial potential for improving employee health and well-being. The aim of this pilot study was to determine the effect of a worksite nutrition intervention on improving well-being.

**Methods:**

Thirty-five university employees participated in a 6-week nutrition intervention. The dietary protocol emphasized the daily consumption of greens, beans/legumes, a variety of other vegetables, fruits, nuts, seeds, and whole grains, referred to as a micronutrient-dense, plant-rich diet. Participants were encouraged to minimize the consumption of refined foods and animal products.

**Results:**

Significant improvements in sleep quality, quality of life, and depressive symptoms were found.

**Conclusions:**

Findings reveal that a worksite nutrition intervention is effective at improving sleep quality, quality of life, and depressive symptoms with a projected improvement in work productivity and attendance.

## 1. Introduction

Despite increased work flexibility in several professions across several industries, many people still find themselves spending significant amounts of time at a worksite or engaged in work related at a worksite. As a result, the worksite environment is a prime location for employers to address the overall health and well-being of their employees. While many worksite interventions have traditionally focused on physical health, the national trend continues to move toward a broader approach to overall health that seeks to maintain a balance of physical, mental, and social aspects. As a result, employers are becoming increasingly aware of the link between mental health status and its relation to work productivity, job satisfaction, medical expenses, absenteeism, and presenteeism [1].

Although cardiovascular disease (CVD) continues to be the number one cause of morbidity and mortality worldwide, the mental health of workers is an area of increasing concern for employers. The most common mental health ailment is depression. Employees living with depression reportedly cost an estimated $35.7 billion a year and account for up to 80% of the depression-related loss in work productivity worldwide [2]. Although the utilization of nutrition interventions to improve mental health and quality of life at this point is limited, evidence is very promising [3]. The dietary mechanisms showing the greatest favor are the approaches that result in a reduction of low-grade systemic inflammation [4].

This micronutrient-dense, plant-rich diet (mNDPR), the dietary pattern utilized in this study, has been proven to be safe and effective in clinical applications not only with inflammation reduction [5], but also with weight reduction and blood pressure lowering [6], lipid management [7, 8], glycemic control in diabetes [9], perceptions of hunger [10], precautionary approaches for cancer [11], and overall health and longevity [12].

## 2. Purpose

The purpose of this pilot study was to determine the impact and effectiveness of a mNDPR dietary intervention on employee well-being when administered at the worksite.

## 3. Methods

### 3.1. Participants

Individuals 18–80 years of age with a body mass index (BMI) ≥ 25 who were ready and willing to make a lifestyle change; who were not currently participating in a weight-loss program; taking any medications that could increase medical risk or had weight loss as a primary side effect were eligible to participate. Participants were recruited through electronic messaging, fliers, and website promotion by the Northern Arizona University (NAU) Department of Employee Assistance and Wellness (EAW). The protocol and study design were approved by the NAU Institutional Review Board (IRB) and all participants were provided written informed consent.

A total of 35 employees participated in this pilot study. The mean age was 42.57 years, with a range of 24 to 61 years, and 91.4% were female. In reference to ethnicity, 80% were Caucasian, 8.6% were Hispanic, 5.7% were black, and 5.7% were Native American.

Participants did not receive financial compensation but were eligible for incentives through their worksite wellness program, potentially offsetting the cost of their personal health insurance premiums. Upon completion of each phase of the study, participants were eligible to receive a set number of points for each aspect that corresponded payments to offset insurance premiums. For full credit of points, participants were required to successfully complete the entire study. Drawings for gift cards were also made at the weekly meetings to provide an added incentive for attendance.

### 3.2. Study Design

Participants attended a 12-hour immersion prior to the commencement of the intervention. The 12 hours consisted of eight segments/lectures over two days in a seminar style format. The immersion program sessions explained in detail the foundational principles of the intervention with significant portions dedicated to the practical application as well as questions and answers throughout each session. Participants then attended 1-hour weekly group meetings to receive instruction, support, encouragement, a cooking demo, food tasting, and to interact with other volunteers and the research team, each of the subsequent six weeks (excluding week 3–spring break) of the pilot study.

The micronutrient-dense, plant-rich (mNDPR) dietary protocol, also called a Nutritarian diet, consisted of foods commonly found in local grocery stores and supermarkets. This was a vegetable-based diet that emphasized daily consumption of greens, beans/legumes, a variety of other vegetables, fresh or frozen fruits, nuts, seeds, and whole grains. Participants were encouraged to minimize consumption of refined grains, vegetable oils, processed foods, and to limit animal products to eight ounces per week or less. The Nutritarian diet does not generally emphasize portion sizes or a specific caloric intake. It is designed to be micronutrient-rich (particularly high in plant-derived phytochemicals), nutritionally complete, and hormonally favorable. It avoids high glycemic-index carbohydrates that can elevate serum insulin levels.

To offer a memorable guide for daily food choices, participants were provided with the acronym GBOMBS + T. The acronym represents greens, beans, onions, mushrooms, berries, seeds, and nuts plus tomatoes.

The use of a multivitamin containing vitamin B12, iodine, zinc, and vitamin D was also encouraged as well as a relatively small amount of EPA-DHA from algae, to assure comprehensive nutrient adequacy, considering the low amount of animal products recommended by this program.

Participants were advised to continue with their current exercise habits and not to drastically alter their physical activity habits during the intervention period. Participants were encouraged to inform their primary health-care provider of their participation in this intervention. Participants were provided contact information of worksite health service providers in case they need these services.

### 3.3. Outcome Measures

One week prior to the 12-hour immersion and one week following the six weeks of intervention, participants completed a medical history and lifestyle questionnaire, the Pittsburgh Sleep Quality Index (PSQI), the Quality of Life Index (QLI), and the Beck Depression Inventory–II (BDI-II). In addition, anthropometric measurements were performed including height, weight, waist circumference, hip circumference, waist-hip ratio (WHR), and blood pressure. Attendance was measured in total hours of attendance throughout the entire study. Compliance was measured at the weekly meetings by making participants complete a self-reporting survey that recorded the percentage of the food and meals consumed that adhered to this intervention's dietary guidelines. Biometric outcomes have been reported elsewhere [7].

The Pittsburgh Sleep Quality Index (PSQI) is a 19-question self-rated instrument designed to assess the sleep quality during the previous month. These 19 questions assess a wide range of factors known to influence the quality of sleep and are weighted equally on a 0–3 scale. The scores are then added up and range from 0 to 21 with a score of 5 or higher indicates poor sleep quality and the higher scores indicative of worse sleep quality. It takes an average of 5–10 minutes for participants to complete and 5 minutes to score [13].

The Quality of Life Index (QLI) Generic Version–III was utilized to measure quality of life in terms of satisfaction with life [14]. Ferrans describes quality of life as “a person's sense of well-being that stems from satisfaction or dissatisfaction with the areas of life that are important to him/her,” [15]. The QLI scores reflect the respondents' satisfaction with the aspects of life they value. Items that are rated as more important have a greater impact on scores than those of lesser importance. The Beck Depression Inventory–II (BDI-II) consists of 21 items arranged in increasing severity about a particular symptom of depression such as hopelessness and irritability, cognitions such as guilt or feelings of being punished, as well as physical symptoms such as fatigue, weight loss, and lack of interest in sexual activity. The BDI utilizes a different Likert type response scale for each item representing from not present (0), more than usual (1), much more than usual (2), to all of the time (3). Each of the four response options increases in severity ranging from not present (0) to more severe (3), and the possible total score for the entire inventory ranges between 0 and 63. The total score cutoffs use the following diagnostic terms to classify individuals as depressed start with minimal depression (scores of 0–13), mild depression (scores of 14–19), moderate depression (scores of 20–28), and severe depression (scores of 29–63). Higher scores from the BDI represent more severe symptomatology for depression [16].

Participants wore a wGT3X-BT monitor, a wrist-worn triaxial accelerometer, from ActiGraph (Pensacola, FL, USA) during a 7-day portion of the intervention, to rule out physical activity as a confounder. The measures included daily axis counts, average counts per minute/per day, and average steps per day to capture and record continuous, high-resolution physical activity, and sleep/wake information. This information allows for the tracking of activity and rest periods for each 24-hour period.

### 3.4. Statistical Analysis

SYSTAT software version 13.1 was used for all analyzes (Systat Software, Inc. San Jose, CA, USA). An alpha of 0.05 was used for all determinations of significance. To determine whether significant changes in the outcome measures occurred across time, paired sample *t*-tests and Wilcoxon signed-ranks tests were used. Wilcoxon tests were used if the dependent variable did not have a normal distribution at either or both times sampled, based on the Shapiro–Wilk test. Change variables were created for each of the outcome variables to examine the association between change and attendance, diet compliance, and activity level. Neither the attendance, activity, nor compliance variables had a normal distribution based on the Shapiro–Wilk test; therefore, associations were measured using Spearman correlations.

## 4. Results

### 4.1. Sleep-Related Variables

There was a significant change in PSQI scores after the intervention. There was a significant change in PSQI scores after the intervention, based on both a paired sample *t*-test. (*z*=−2.663,  *p*=0.008). Shapiro–Wilk univariate normality tests indicated that PSQI scores were not normally distributed at the time of the posttests.

There was also a significant change in total hours of sleep, based on both a paired sample *t*-test (*z*=2.683,  *p*=0.007). Shapiro–Wilk univariate normality tests indicated that total hours of sleep were not normally distributed at the time of the pretests (Table 1).

### 4.2. Quality of Life Variables

Based on both a paired sample *t*-test (*t*(33) = −3.303, *p*=0.002) test and a Wilcoxon Signed Ranks test (*z*=2.904,  *p*=0.004), there was a significant change in the overall Quality of Life Index (QLI) scale after the intervention. Shapiro–Wilk univariate normality tests indicated that overall QLI was not normally distributed at the time of the pretests.

The QLI scale had four subscales: health, socioeconomic, psychological, and family. Except for the subscale related to family, all subscale scores were normally distributed at pretest and posttest. The QLI-Family subscale was not normally distributed at either pretest or posttest.

There were significant changes in QLI-Health (*t*(33) = −4.678, *p* < 0.001) and in QLI-Psychological (*t*(33) = −4.132, *p* < 0.001). QLI-Family scores also changed over time, based on a paired samples *t*-test (*t*(32) = −2.083, *p*=0.045) and a Wilcoxon signed ranks test (*z*=2.253,  *p*=0.024) (Table 1).

### 4.3. Beck Depression Variables

Both *t*-test (*t*(32) = −3.303, *p*=0.002) and Wilcoxon signed-ranks test (z=−3.924, *p* < 0.001) indicated a significant change in depression scores after the intervention. Shapiro–Wilk univariate normality tests indicated that BDI scores were not normally distributed either the pretest or the posttest (Table 1).

### 4.4. Impact of Attendance, Dietary Compliance, and Physical Activity

The overall compliance and attendance variables were not normally distributed; thus, Spearman rank correlations were selected to examine the relationship between change in the outcome variables and attendance and dietary compliance. The only significant correlation shown was between change in socioeconomic, QLI, and attendance (Table 2).

There was no correlation between the physical activity measures and changes in the outcome measures (Table 3).

## 5. Discussion

The overall improvements in the various cardiovascular risk factors were significant and were previously reported [7]. In addition, the corresponding improvements in overall well-being seem to also have occurred due to the broad variety of nutrients in the mNDPR diet as well as a subsequent reduction in refined foods and animal products. These findings suggest that this diet style can have a positive impact on overall well-being. The micronutrients in the mNDPR do not merely have anticancer and longevity promoting effects; they also exert neuroprotective effects in experimental models of psychiatric disorders [17]. As a natural part of the human diet, these micronutrients are necessary for cell signaling pathways within the brain. This means that the presence or absence of these micronutrients affect brain function and the development of brain pathology.

Specifically, in relation to depression, our findings parallel those of Akbaraly et al. that show a dietary pattern, with fried food, sweetened desserts, processed meat, and refined grains, has been associated with depression, and the consumption of whole natural foods has been shown to be strongly protective [18]. In addition, Sánchez-Villegas et al. [19] found that fast food and commercial baked goods are linked to depression in a dose-dependent manner. Their findings revealed that consumers of fast food, compared to those who eat little or none, are 51 percent more likely to develop depression, and the more you consume, the greater the risk is. This depression-inducing dietary pattern appears to be associated with high glycemic carbohydrates, of which this intervention sought to minimize. In addition, Gangwisch et al. [20] found a dose-dependent effect from high glycemic load foods (white flour and sweetening agents) and depression. Many of our study participants noted a link between eating sugary foods and feeling down the next day, which is consistent with findings from the Women's Health Initiative Observational Study (WHIOS) of nearly 70,000 women. The WHIOS found that foods higher on the glycemic index, including those rich in refined grains and added sugar, were associated with greater odds of depression; however, the researchers found eating high-fiber foods such as whole grains, whole fruits, and vegetables lowered their odds.

Our findings also revealed that many participants were pleased with weight loss, how their clothes fit, their improved mood, and with their accomplishments which may be correlated with an overall increase in happiness, sleep quality, and quality of life. Participants also expressed satisfaction with learning how to shop, cook, and prepare micronutrient-dense meals for themselves in a way that helped them feel good physically and emotionally. The improvements in well-being appear to be synergistically related to cardiometabolic improvements and the associated benefits from an increase in micronutrient consumption.

## 6. Limitations

Possible limitations for this pilot study include no control group, sample size, the majority of the participants were female, sample characteristics, and the demographic region.

## Figures and Tables

**Table 1:** Pre- and postintervention measures of central tendency and variability.

	Preintervention mean and SD	Postintervention mean and SD	Preintervention median and interquartile range	Postintervention median and interquartile range
Pittsburgh Sleep Quality Index (PSQI)	6.676 4.353	3.364 2.922	6.000 4.000	4.000 4.000
Total hours of sleep	6.897 1.127	7.309 0.963	7.000 2.000	7.500 1.000
Quality of Life Index (QLI–Generic-III)–Overall	19.920 4.387	22.457 4.110	20.880 6.850	23.130 5.900
Quality of Life Index (QLI–Generic-III)–Health	19.744 4.517	23.336 3.665	19.810 7.080	23.880 5.690
Quality of Life Index (QLI–Generic-III)–Psychological	19.326 4.893	22.463 4.572	20.890 7.140	22.925 6.00
Quality of Life Index (QLI–Generic-III)–Socioeconomic	21.910 3.949	22.722 4.247	22.825 6.000	23.215 5.79
Quality of Life Index (QLI–Generic-III)–Family	19.065 7.256	21.434 6.615	19.900 9.052	21.630 10.00
Beck Depression Inventory II (BDI-II)	10.455 6.865	5.303 4.707	9.000 5.750	4.000 6.250

**Table 2:** Spearman correlations between change in outcome variables and overall attendance and overall dietary compliance.

	Overall attendance at meetings	Overall compliance
Overall compliance	0.129	—
Daily axis counts	−0.028	0.067
Average counts per minute/per day	−0.028	0.067
Average steps per day	−0.108	0.119
Pittsburgh Sleep Quality Index (PSQI)	0.181	0.116
Total hours sleep	−0.043	−0.204
Quality of Life Index (QLI–Generic-III)–Overall	−0.213	0.025
Quality of Life Index (QLI–Generic-III)–Health	0.065	0.058
Quality of Life Index (QLI–Generic-III)–Psychological	−0.093	−0.093
Quality of Life Index (QLI–Generic-III)–Socioeconomic	0.397	−0.038
Quality of Life Index (QLI–Generic-III)–Family	−0.153	−0.148
Beck Depression Inventory II (BDI-II)	0.224	0.253

**Table 3:** Spearman correlations between change in outcome variables and measured exercise levels.

	Daily axis counts	Average counts per minute/day	Average step count per day
Overall attendance at meetings	−0.054	−0.054	−0.117
Overall compliance	0.085	0.085	0.113
Pittsburgh Sleep Quality Index (PSQI)	0.168	0.168	0.187
Total hours sleep	0.154	0.154	0.003
Quality of Life Index (QLI–Generic-III)–Overall	−0.002	−0.002	−0.071
Quality of Life Index (QLI–Generic-III)–Health	0.066	0.066	0.156
Quality of Life Index (QLI–Generic-III)–Psychological	0.097	0.097	0.179
Quality of Life Index (QLI–Generic-III)–Socioeconomic	−0.117	−0.117	−0.108
Quality of Life Index (QLI–Generic-III)–Family	−0.025	−0.025	0.021
BDI-II (Beck Depression Inventory–II)	0.064	0.064	0.151

## Data Availability

The datasets generated during this study are available from the corresponding author upon reasonable request.
